# Helical tomotherapy in the treatment of pediatric malignancies: a preliminary report of feasibility and acute toxicity

**DOI:** 10.1186/1748-717X-6-102

**Published:** 2011-08-26

**Authors:** Latifa Mesbah, Raúl Matute, Sergey Usychkin, Immacolata Marrone, Fernando Puebla, Cristina Mínguez, Rafael García, Graciela García, César Beltrán, Hugo Marsiglia

**Affiliations:** 1 Radiotherapy Department, Instituto Madrileño de Oncología (Grupo IMO), 7 Plaza Republica Argentina, Madrid, 28002, Spain; 2 Breast Cancer Unit, Institut de Cancerologie Gustave Roussy, 39 Rue Camille Desmoulins, Ville Juif, Paris, 94805, France; 3 University of Florence, 14 Via della Mattonaia, Florence, 50121, Italia

**Keywords:** Helical Tomotherapy, Intensity-Modulated Radiation Therapy, pediatric malignancies, feasibility, acute toxicity

## Abstract

**Background:**

Radiation therapy plays a central role in the management of many childhood malignancies and Helical Tomotherapy (HT) provides potential to decrease toxicity by limiting the radiation dose to normal structures. The aim of this article was to report preliminary results of our clinical experience with HT in pediatric malignancies.

**Methods:**

In this study 66 consecutive patients younger than 14 years old, treated with HT at our center between January 2006 and April 2010, have been included. We performed statistical analyses to assess the relationship between acute toxicity, graded according to the RTOG criteria, and several clinical and treatment characteristics such as a dose and irradiation volume.

**Results:**

The median age of patients was 5 years. The most common tumor sites were: central nervous system (57%), abdomen (17%) and thorax (6%). The most prevalent histological types were: medulloblastoma (16 patients), neuroblastoma (9 patients) and rhabdomyosarcoma (7 patients). A total of 52 patients were treated for primary disease and 14 patients were treated for recurrent tumors. The majority of the patients (72%) were previously treated with chemotherapy. The median prescribed dose was 51 Gy (range 10-70 Gy). In 81% of cases grade 1 or 2 acute toxicity was observed. There were 11 cases (16,6%) of grade 3 hematological toxicity, two cases of grade 3 skin toxicity and one case of grade 3 emesis. Nine patients (13,6%) had grade 4 hematological toxicity. There were no cases of grade 4 non-hematological toxicities. On the univariate analysis, total dose and craniospinal irradiation (24 cases) were significantly associated with severe toxicity (grade 3 or more), whereas age and chemotherapy were not. On the multivariate analysis, craniospinal irradiation was the only significant independent risk factor for grade 3-4 toxicity.

**Conclusion:**

HT in pediatric population is feasible and safe treatment modality. It is characterized by an acceptable level of acute toxicity that we have seen in this highly selected pediatric patient cohort with clinical features of poor prognosis and/or aggressive therapy needed. Despite of a dosimetrical advantage of HT technique, an exhaustive analysis of long-term follow-up data is needed to assess late toxicity, especially in this potentially sensitive to radiation population.

## Background

Radiation therapy is an integral part in the treatment of 40-60% of childhood cancer patients [1]. Although many childhood malignancies are cured, the acute toxicity of therapy and significant late treatment effects make these cancers a substantial burden for patients, their families, and society [2]. Therefore, the goal of modern strategies is not only to improve cancer cure rate, but also to decrease adverse sequelae of treatment. The use of modern radiotherapy techniques may, potentially, decrease the incidence and severity of radiation toxicity.

Intensity-Modulated Radiation Therapy (IMRT) has shown to be a safe and effective treatment modality for adult cancer patients. This radiotherapy delivery technique has proven capability to create highly conformal dose distributions allowing to escalate dose in target volume and to spare adjacent organs at risk [3,4]. While IMRT is widely used as a standard of care for many adult cancers patients, this technique has been used less frequently in childhood cancer patients, for several reasons, such as a potentially augmented risk of carcinogenesis due to increased volume of normal tissues receiving low-dose radiation.

Helical Tomotherapy (HT) is a novel highly precise IMRT technique with image-guidance using megavoltage computed tomography (MVCT) that actually is used by more than 150 institutions around the word. In Spain, it was implemented for the first time in 2006, at the Instituto Madrileño de Oncología (Grupo IMO), which is a referral center of pediatric radiation oncology in the country. In this article we report our initial experience of HT in the treatment of pediatric malignancies, focused on analysis of tumor response and acute radiation toxicity. A critical review of published studies of IMRT and HT in the treatment of pediatric cancer patients is also presented.

## Methods

From April 2006 through May 2010, 66 consecutive children younger than 14 years old underwent HT at the Tomotherapy Unit of the Grupo IMO in the context of multidisciplinary national and international treatment protocols. All the patients were treated with curative intent, including those who had recurrent disease. Two patients previously had received external beam radiation therapy, one of them underwent reirradiation for local recurrence of rhabdomyosarcoma (RMS), and the other patient received reirradiation for spinal recurrence of medulloblastoma. All patients were referred to our center from their local radiotherapy departments due to inability of conventional radiotherapy techniques to comply with dose restrictions in critical organs.

**Figure 1:**
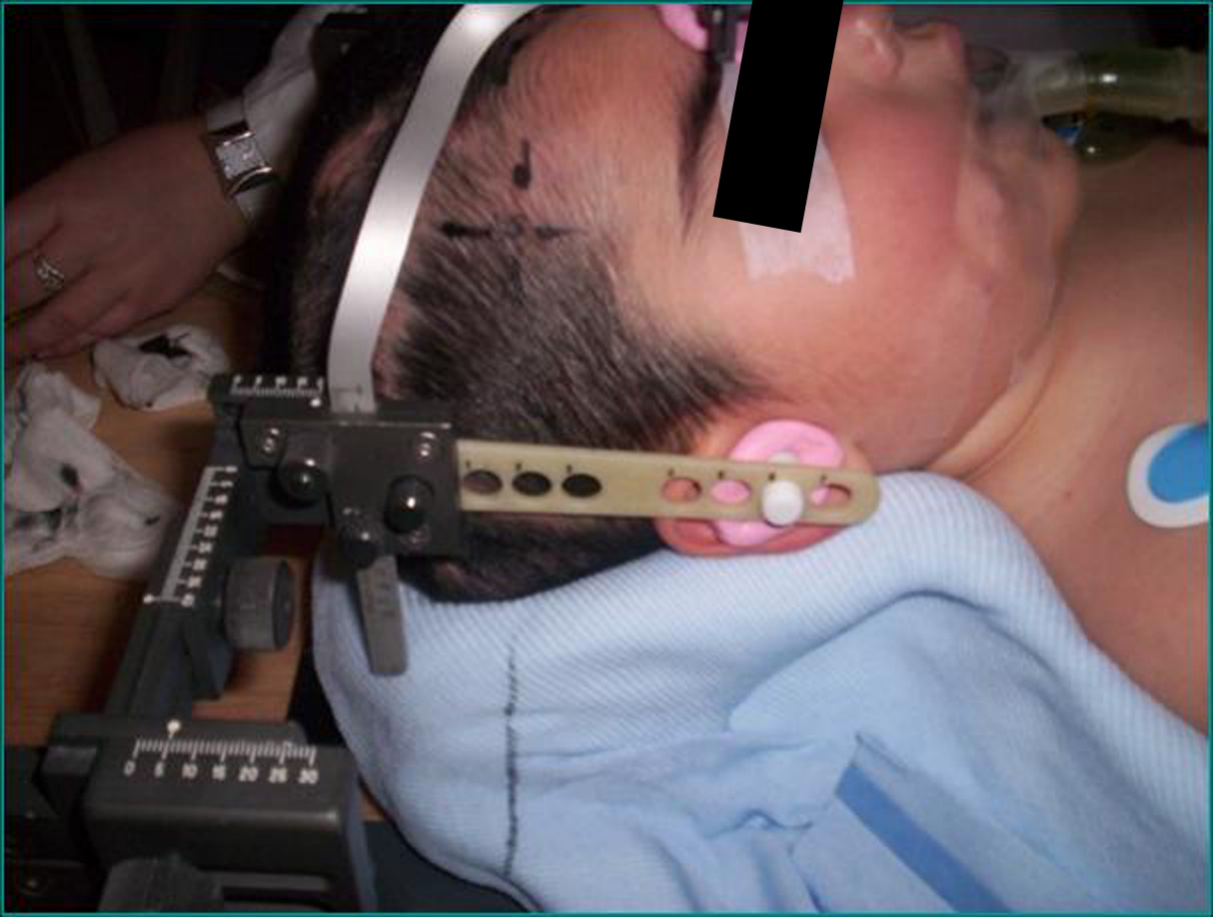
**"Home-made" non-invasive stereotactic frame**.

Individual immobilization was employed in all cases. Depending on the site of the treatment, a customized alpha-cradle mould was used for thoracic and abdominopelvic tumor sites, whereas a 'home-made' non-invasive stereotactic frame system was used for head and neck tumors (Figure 1).

Target volumes were defined using only computed tomography images in 23 patients. In 43 patients co-registration of 18-fluorodeoxyglucose positron emission tomography and/or magnetic resonance images with computed tomography images was used. Target volumes and organs at risk were contoured on a Pinnacle™ workstation version 8.0 (Philips Radiation Oncology Systems, Fitchburg, WI, USA) and defined according to the criteria of the International Commission of Radiation Units and Measurement [5,6]. As a rule 3 to 5 mm CTV to PTV margins were applied. Data sets and structures were transferred to the Tomotherapy treatment planning system (Tomotherapy Inc., Madison, WI) to perform inverse treatment planning. The planning goal was to deliver the prescription dose to at least 95% of the PTV. The dose constraints for organs at risk (OARs) were mainly those reported in of the National Cancer Institute Physician Data Query [7]. Dose volume histograms for PTVs and OARs were recorded from the dosimetric charts. Homogeneity index was calculated dividing the maximal PTV dose by the prescription dose; the coverage index was calculated dividing the minimum PTV dose by the prescription dose. Both indexes were calculated accordingly to the recommendations established for evaluating tomotherapy treatment plans [8].

All treatments were delivered by a Helical TomoTherapy™ HiArt™ II system treatment unit. Daily MVCT acquisitions were performed for all patients to detect set-up deviations and to correct them. All patients were treated with once-daily fractions of 1.5-2 Gy, except for one child with medulloblastoma who received twice-daily fractionated radiotherapy.

All patients were examined at least weekly during treatment. The acute and subacute toxicity was defined and graded according to the RTOG criteria. After the radiation therapy, all the patients underwent follow-up examinations at 1, 3, 6 months after treatment and then yearly.

### Statistical analysis

Univariate analysis was performed to test the association between several clinical and treatment characteristics and ≥ grade 3 acute toxicity. The t test or the non-parametric Mann-Whitney test (if the normal distribution assumption was not fitted) was used for quantitative variables and a chi-square test for qualitative variables. For the multivariate analysis a regression logistic was performed. Two-tailed p-values < 0.05 were considered to be statistically significant. Analyses were performed using SPSS version 15 (SPSS Inc., Chicago, IL).

## Results

The median age at HT treatment was 5 years (range 1-14 years); 20 patients (30%) were 3 years old or younger. Patient characteristics are summarized in Table 1. The most common tumor sites were central nervous system (57%), abdomen (17%) and thorax (6%). The most prevalent histological types were medulloblastoma (16 patients), neuroblastoma (9 patients) and rhabdomyosarcoma (7 patients). 52 patients were treated for primary disease while 14 patients were treated for recurrence. The majority of the patients (72%) received neoadjuvant or concomitant chemotherapy. The median administered radiation dose was 51 Gy (range 11 Gy - 70 Gy).

**Table 1:** Patients characteristics

Characteristics				n (%)
Gender	Male			36 (55%)
	Female			30 (45%)
	
		Medulloblastoma		16 (24%)
		Ependymoma and ependymoblastoma		8 (12%)
		Glioma		7 (11%)
	CNS	Pineoblastoma		2 (3%)
		Teratoid/Rhabdoid tumor		2 (3%)
		Germinal tumor		1 (1%)
		Choroid plexus tumor		1 (1%)
		Craniopharyngioma		1 (1%)
	
Tumor site/histology	Abdomen	Neuroblastoma		7 (11%)
		Nephroblastoma		2 (3%)
		Rhabdomyosarcoma		1 (1%)
		Clear cell sarcoma		1 (1%)
	
	Thorax	Ewing sarcoma		1 (1%)
		Hodgkin lymphoma		1 (1%)
		PNET (Askin's) tumor		1 (1%)
		Rhabdomyosarcoma		1 (1%)
	
	Pelvis	Rhabdomyosarcoma		2 (3%)
		Ewing sarcoma		1 (1%)
		PNET tumor		1 (1%)
	
	Other sites	Orbit	Melanoma	1 (1%)
			Rhabdomyosarcoma	1 (1%)
			PNET tumor	1 (1%)
		Spine	Neuroblastoma	2 (3%)
		Skull base	Chordoma	1 (3%)
		Oropharynx	Rhabdomyosarcoma	1 (1%)
		Extremity	Rhabdomyosarcoma	1 (1%)
		Sub- and supradiaphragmatic Hodgkin lymphoma		1 (1%)

Sedation with inhalation of sevoflurane during radiotherapy session was necessary in 41 patients (62%). Median age of these patients was 4 years (range 1-9 years). They were treated with craniospinal irradiation (n = 16, 40%) and extended target volumes irradiation in thorax and abdominal (n = 8, 20%) which were main indications for sedation. It was well tolerated without severe side-effects and was associated with fast recovery after treatment. General anesthesia with intubation was not necessary.

Acute toxicity data is summarized in Table 2. In 81% of cases grade 1 or 2 acute toxicity was observed. There were 11 cases (16,6%) of grade 3 hematological toxicity, two cases of grade 3 skin toxicity and one case of grade 3 emesis. Nine patients (13,6%) had grade 4 hematological toxicity. We have not seen any case of grade 4 non-hematological toxicity.

**Table 2:** Rate of acute toxicity by grade

	Toxicity (Grade)				
	
	1	2	3	4	total
Hematological	8	5	11	9	33 (29%)
Skin	30	3	2	0	35 (31%)
Gastrointestinal	13	20	1	0	34 (30%)
SNC	3	1	0	0	4 (3%)
Ear	1	1	0	0	2 (2%)
Eye	4	2	0	0	6 (5%)
Total	59 (51%)	34 (30%)	13 (11%)	9 (8%)	114 (100%)

Actual daily treatment was not recorded during treatment sessions. However it can be estimated approximately based on daily treatment practice of our department. In analyzed cases of pediatric malignancies daily treatment time was composed of time required for patient set-up and anesthesia inside the treatment room, time of MVCT acquisition, time of review/match and applying couch correction inside the treatment room, actual radiation delivery time and waiting time of patient recovery (from end of irradiation until the patient is awake) from anesthesia. Time of MVCT acquisition and actual radiation delivery time are factors that mostly influence time of treatment session. It's known that in helical tomotherapy these parameters strongly depend on the longitudinal extension of irradiated volume and as well as on selected MVCT slice thickness. For example, in case of craniospinal irradiation typical time of MVCT acquisition in our department is about 300-500 seconds. Time needed for review and match of images is no more than 1-3 minutes. Radiation delivery time was recorded for each patient in treatment chart. It varied from 158 to 1991 seconds and median was 390 seconds thus showing strong dependence on the extension of treated volume. Radiation delivery time for selected "challenging" tumor sites is presented in Table 3. Patient set-up and anesthesia requirements prolong daily treatment time for about 5-10 min and generally do not compromise treatment time frame of these patients.

**Table 3:** Target volume coverage and homogeneity indices for selected challenging cases

Tumor site	Histology (number of cases)	Target volume	Prescribed dose, Gy	Mean PTV dose, Gy*	Coverage Index^§^	Homogeneity Index^§^	Irradiation time (sec) †
CNS (craniospinal irradiation)	Medulloblastoma (16)	Whole brain	23,4	23,98 ± 0,17	0,78 (0,53-0,95)	1,10 (1,07-1,21)	912,7 (367,4 - 1991,2)
			36,0	36,96 ± 0,15	0,74 (0,47-0,90)	1,10 (1,08-1,12)	
		Cribriform plate	23,4	23,88 ± 0,07	0,86 (0,75-0,95)	1,07 (1,04-1,09)	
			36,0	36,86 ± 0,30	0,79 (0,62-1,00)	1,07 (1,06-1,09)	
		Spinal canal	23,4	23,90 ± 0,16	0,87 (0,73-0,91)	1,07 (1,06-1,09)	
			36,0	36,82 ± 0,45	0,90 (0,78-1,00)	1,07 (1,06-1,13)	
		Tumor bed	54,0	55,06 ± 0,49	0,81 (0,57-0,98)	1,05 (1,02-1,13)	
CNS	Glioma (7)	Tumor/tumor bed	45,0-59,4	45,18-60,76	0,89 (0,81-0,98)	1,04 (1,02-1,06)	328,0 (211,8 - 957,0)
Abdomen	Neuroblastoma (7)	Tumor bed	21,0	21,34 ± 0,13	0,85 (0,48-0,94)	1,07 (1,03-1,08)	256,8 (158,8 - 293,2)
Thorax	Rhabdomyosarcoma (1)	Right pleura	50,4	50,11 ± 0,98	0,84	1,02	730,3
	PNET (Askin's tumor) (1)	Hemithorax	14,40	14,83 ± 0,19	0,89	1,09	554,1
		GTV	48,60	49,87 ± 0,79	0,74	1,06	
	Ewing sarcoma (1)	Hemithorax	14,00	14,38 ± 0,24	0,77	1,07	519,0
		Tumor	48,00	49,29 ± 0,22	0,90	1,08	
		Met L2-S1	48,00	49,22 ± 0,15	0,92	1,05	
Pelvis	Rhabdomyosarcoma (1)	Inguinal nodes	41,40	41,94 ± 0,59	0,91	1,05	327,2
		Tumor bed	50,40	51,10 ± 0,62	0,65	1,04	
Total lymphatic irradiation	Hodgkin lymphoma (1)	Liver, spleen, total lymphatic	12,00	12,46 ± 0,25	0,76	1,07	538,2
		Total lymphatic	21,00	21,74 ± 0,22	0,74	1,09	
Orbit	PNET (1)	Tumor	48,60	49,40 ± 0,86	0,55	1,05	344,1
	Rhabdomyosarcoma (1)	Tumor bed	50,40	51,93 ± 0,83	0,94	1,07	479,8
	Melanoma (1)	Tumor bed	50,40	51,16 ± 0,42	0,98	1,08	329,6

* Data are presented as mean ± SD or as a range of mean PTV dose

^§ ^Data are presented as median (range) for groups and as single values for individual cases

† Data are presented as irradiation time for the phase of treatment with longest irradiation time and as median (range) for groups

In a great proportion of patients (39%) we were able to deliver radiation to extended volumes without field junctions: craniospinal irradiation was performed in 23 patients; two patients underwent hemithorax irradiation, one for thoracic Askin's tumor and the other for thoracic Ewing sarcoma; in one case of advanced Hodgkin lymphoma the patient received near total lymphatic irradiation.

**Figure 2:**
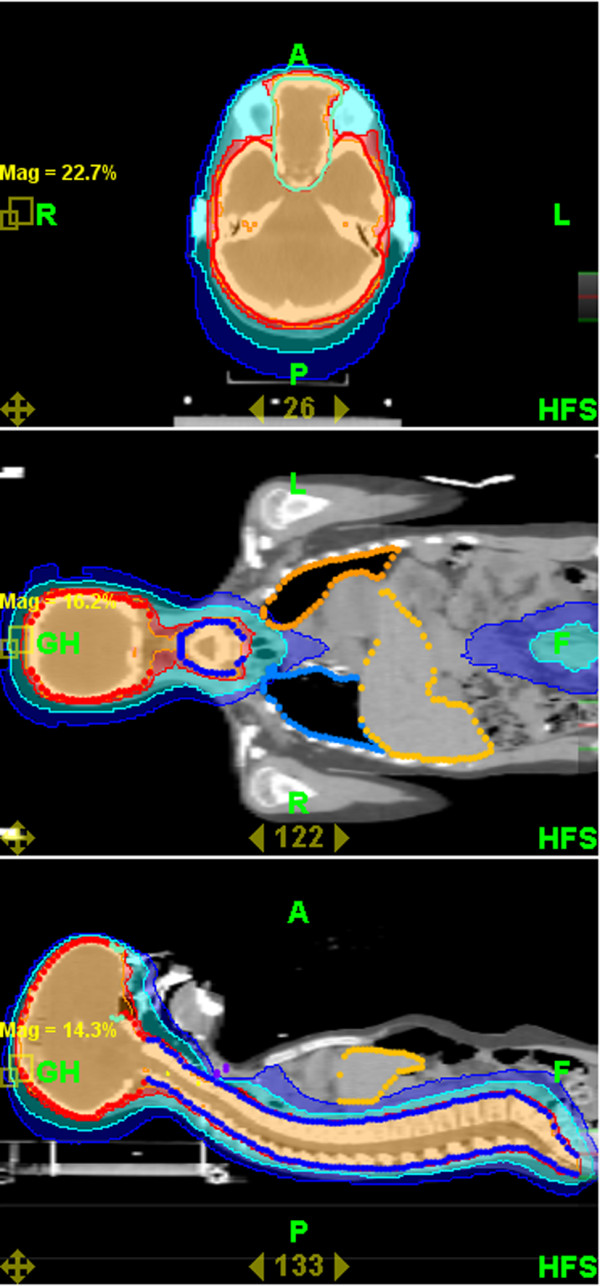
**Dose distribution for craniospinal irradiation**.

**Figure 3:**
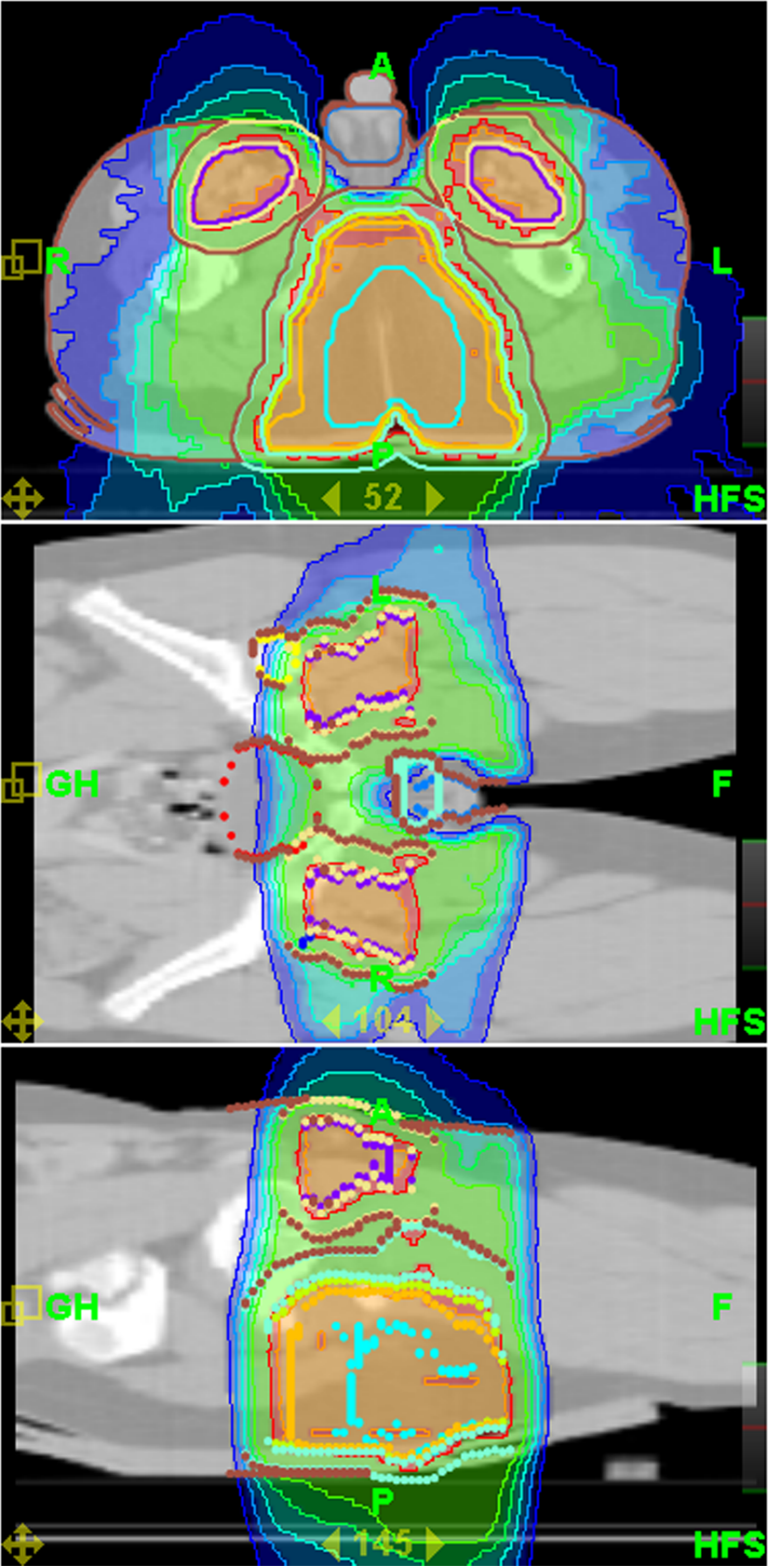
**Dose distribution for perineal rhabdomyosarcoma**.

Mean coverage index for entire group of patients and all PTVs was 0,82 ± 0,13. Mean homogeneity index was 1,07 ± 0,02. Mean PTV doses, coverage and homogeneity indexes for selected challenging cases or groups of patients are presented in Table 3. Even for challenging cases of craniospinal irradiation and extended thoracic and abdominal volumes irradiation coverage and homogeneity of delivered dose were acceptable. Mean doses for selected OARs are presented in Table 4. It shows that substantial sparing of critical structures was achieved in all patients although major variability in OARs mean doses in this very heterogeneous patient population is evident. In Figures 2 and 3 examples of treatment plan for medulloblastoma and perineal rhabdomyosarcoma with metastases to inguinal nodes are presented.

**Table 4:** Mean doses in OARs for selected tumor sites

Tumor site	Craniospinal irradiation		Intracranial lesions	Abdominal lesions	Thoracic lesions	Pelvic lesions
	23,4 Gy (CSI) + 54 Gy (tumor bed)	36 (CSI) + 54 Gy (tumor bed)	50,4 - 54 Gy	21 Gy	48 - 50,4 Gy	50,4 - 63 Gy
Normal brain	-	-	14,99 ± 6,34	-	-	-
Chiasm	-	-	36,24 ± 9,27	-	-	-
Eyes	12,81 ± 5,38	19,81 ± 4,43	6,25 ± 3,17	-	-	-
Lens	4,56 ± 3,17	6,59 ± 0,99	3,73 ± 1,2	-	-	-
Cochleae	28,94 ± 9,45	42,42 ± 6,06	-	-	-	-
Optic nerves	25,37 ± 1,53	37,23 ± 4,93	22,38 ± 12,16	-	-	-
Brainstem	47,40 ± 4,18	49,73 ± 2,64	33,17 ± 17,55	-	-	-
Kidneys	8,79 ± 2,25	11,68 ± 4,34	-	8,73 ± 1,19	-	-
Liver	5,99 ± 0,84	9,11 ± 1,15	-	7,44 ± 1,66	20,23 ± 10,20	-
Lungs	7,27 ± 1,31	10,82 ± 2,64	-	3,25 ± 0,87	8,61 ± 5,37	-
Heart	6,50 ± 2,15	11,74 ± 1,04	-	-	16,27 ± 13,38	-
Spinal cord	-	-	-	20,13 ± 3,78	46,73 ± 2,97	-
Rectum	-	-	-	-	-	32,60 ± 14,47
Urinary bladder	-	-	-	-	-	30,83 ± 21,22
Femoral heads	-	-	-	-	-	14,23 ± 11,63

On the univariate analysis, total dose and craniospinal irradiation were associated significantly with toxicity grade 3 or more, whereas age and chemotherapy were not (Table 5). On the multivariate analysis, craniospinal irradiation was the only significant independent risk factor for grade 3/4 toxicity.

**Table 5:** Univariate analysis for factors associated with ≥ grade3 acute toxicity

Characteristic		Grade 0-2	Grade 3-4	P value
Total dose*		43,1 (15,4)	52,0 (7,6)	0,005^§^
Age^§^		5,4 (+/- 3,1)	7,1 (+/- 4,2)	0,12
Craniospinal irradiation^†^	Yes	8 (18%)	16 (78%)	< 0,001
	No	36 (82%)	6 (27%)	
Chemotherapy^†^	Yes	33 (79%)	19 (86%)	0,52
	No	9 (21%)	3 (14%)	

* Asymmetric distribution verified by Kolmogorov-Smirnov test. Mann-Whitney test performed.

^§ ^Chi-square test.

^† ^t-test

While at present follow-up time is not sufficient (median 15 months; range 2-59 months) for reliable conclusions of survival, the tumor response of 51 patients could be analyzed: in 30 patients (59%) a complete response was obtained, in 5 patients (9%) a partial response, 7 patients (11%) showed stabilization and 5 patients (9%) died due to progressive disease. It's remarkable that actually seven patients with primary rhabdomyosarcoma are alive and free from local or distance relapse of disease.

## Discussion

Helical Tomotherapy is a radiation delivery technique, which is able to create highly conformal dose distributions in target volume. HT was designed as an integrated system for volumetric IGRT and IMRT [9]. Reproducibility of patient positioning is especially important in highly conformal radiotherapy techniques such as HT. The use of daily pretreatment imaging with MVCT allows to reduce the PTV margins and thereby to reduce the amount of normal tissues receiving high doses [10]. That in turn may lead to reduced rate of the long-term side effects. It also allows monitoring of changes in target volumes or patient anatomy during the treatment course, i.e. an adaptive radiotherapy. In addition, the possibility of daily deformable dose registration potentially permits to obtain a true representation of the dose delivered to the patient throughout the course of treatment.

This study aimed to address the feasibility of HT in the treatment of various pediatric tumor sites. We present a very heterogeneous group of young children with tumors that are extremely difficult to treat with conventional radiotherapy techniques. HT allowed us to perform reirradiation in challenging tumor sites that could not be performed safely before. HT was easily administered, even for very young children who required anesthesia. No anesthesia related toxicity associated with prolongation of treatment session time due to MVCT imaging verification was noted.

In all cases HT generated clinically acceptable plan with highly conformal dose distribution and sufficient avoidance of OARs. The analysis of acute toxicities demonstrated that, except for one case of grade 3 gastrointestinal and two cases of grade 3 skin toxicity, no grade 4 non-hematological toxicities were found. This noticeable low rate of acute toxicity deserves attention, since in our study we included highly selected pediatric patient population with clinical features of poor prognosis and/or aggressive therapy needed. For example, 30% of patients were very young (3 years old or less), in 39% of patients large volumes of normal tissues were irradiated, some patients had tumors close to OARs and/or in some cases tumors were reirradiated. Relatively high radiation doses were prescribed (median 51 Gy) and the majority of patients (72%) also received chemotherapy.

In our series, the unique significant factor associated with high degree of hematological toxicity was craniospinal irradiation. In accordance with usual practice, we included all vertebral bodies in the craniospinal irradiation PTV to prevent growth asymmetries. This approach and high load of chemotherapy probably explain observed events of hematological toxicity despite the fact that p-value in the univariate analysis was non-significant.

Due to high heterogeneity and limited follow-up of patient population in this study, we suppose that it would be too risky to make even preliminary conclusions about survival or local control for whole treatment cohort. With more extended follow-up a more reliable analysis of clinical endpoints by tumor sites and histological types will be feasible.

HT is particularly interesting for craniospinal irradiation because of the possibility to irradiate extended volumes without the need for field junctions. Parker et al. demonstrated that HT plan provides superior sparing of critical structures from high doses (> 10 Gy) and excellent target coverage [11]. Similar results had been obtained early by Penagaricano and Bauman [12,13]. Penagaricano et al. recently have published a cohort of 18 children who received craniospinal irradiation with HT, reporting a good local control without any pulmonary radiation-related toxicity [14]. Kunos reported a decrease of hematological acute toxicity and dose to growing vertebrae with HT [15].

HT offers also an advantage for selected patients such as those who require a whole-ventricular irradiation. A dosimetrical study was conducted by Chen et al, comparing 3D conformal radiotherapy (3D-CRT), IMRT, and HT techniques, for six pediatric patients. In this study, a good PTV coverage was achieved in all patients regardless of treatment technique. HT significantly reduced mean dose to the temporal lobes, pituitary gland and chiasm, but not to the brainstem [16].

Another indication HT is a whole abdominal irradiation that involves treatment of large target volumes with complex shape. In this setting HT can be superior to other techniques. Conventional techniques produce inhomogeneous dose distributions due to necessity of kidneys and liver shielding. Rochet and al. explored the potential of HT to lower the dose to kidneys, liver and bone marrow, while covering the peritoneal cavity with a homogeneous dose. HT enabled a very homogeneous dose distribution with excellent sparing of OARs and coverage of the PTV [17].

HT may potentially improve irradiation in Hodgkin's disease (HD). Vlachaki et al. compared the dosimetry of 3D-CRT with HT in pediatric patients with advanced HD. HT decreased mean normal tissue dose by 22% and 20% for right and left breasts respectively, 20% for lung, 31% for heart and 23% for the thyroid gland. Integral dose also decreased with HT by 47% [18].

Fogliata et al. compared HT, RapidArc™ and Intensity Modulated Protons for five challenging pediatric cases in terms of tumor location, anatomical boundary conditions, dose coverage, and tolerance requirements. All techniques sufficiently complied with planning objectives and generated clinically acceptable plans. As expected, protons presented a significant improvement in OARs sparing, at the price of slightly compromised target coverage. The authors conclude that, since the access to proton facilities is still relatively limited in the world, it is of interest to explore advanced photon techniques such as HT and RapidArc™ [19].

Still there is no a randomized study comparing IMRT and the other radiotherapy techniques in the childhood malignancies. The only available data are based on prospective comparative studies or institutional experience that have shown feasibility and in some studies a clinical benefit with the use of the IMRT. In a study of Bhatnagar et al favorable results of IMRT treatment in twenty-two pediatric cancer patients were reported. They reported substantial sparing of surrounding critical structures in very difficult for irradiation cases of cranial, abdominopelvic or spinal tumors [20]. Similar results were demonstrated in a series of 31 patients from Sterzing et al. [21]. Huang et al. reported reduced rate ototoxicity in medulloblastoma patients when the boost dose was delivered by IMRT in comparison to conventional radiotherapy. Thirteen percent of the IMRT Group had grade 3 or 4 hearing loss, compared to 64% of the conventional RT group [22].

Schroeder et al. reported on 22 children with localized intracranial ependymoma treated with IMRT, a three year local control of 68% [23]. These results are similar to those reported by Merchant et al with CRT radiotherapy [24], but no patient developed serious complication in Schroeder series (visual loss, brain necrosis, myelitis, or a second malignancy).

Krasin et al. presented a planning study comparing different conventional photon, electron and IMRT techniques in the treatment of intraocular retinoblastoma. IMRT plans achieved best sparing of the bony orbit. The mean volume of bony orbit treated with IMRT above 20 Gy was 60% in contrast to 90% with the conventional technique [25].

In a study by Wolden et al., 28 patients with head and neck rhabdomyosarcoma were treated with IMRT. The three-year local control was 95% with minimal side effects. One patient developed a local recurrence in treatment field [26]. Curtis et al analyzed the patterns of failure in 19 pediatric patients treated with IMRT for head and neck rhabdomyosarcoma. The 4-year overall survival and local control rates were 76% and 92.9%, respectively. One patient developed a local failure in the high-dose region of the radiation field, there were no marginal failures [27].

Laskar et al presented a cohort of 36 children treated with CRT (n = 17) or IMRT (n = 19) for nasopharyngeal carcinoma. After a median follow-up of 27 months, the 2-year loco-regional control, disease-free and overall survival rate was 76.5%, 60.6%, and 71.3%, respectively. A significant reduction of acute Grade 3 skin, mucosa and pharynx toxicity rate was noted with the use of IMRT. The median time to the development of Grade 2 toxicity was also delayed with IMRT [28].

IMRT and HT allow irradiation of the pediatric tumors with better quality, in particular when the target volume has a complex shape or when is located close to critical structures such as thoracic or pelvic Ewing sarcoma [29].

Another potential advantage of HT in pediatric patients, especially in those with frequent metastatic spread of tumor such as rhabdomyosarcoma and Ewing sarcomas, could be a possibility of simultaneous irradiation of multiple separated lesions. In few pilot studies in adult cancer patients a technical feasibility and clinical efficacy of this technique was demonstrated [30-32].

Although HT can be an elegant way to deliver radiation therapy to target and limit radiation dose to normal structures, this benefit could be achieved at the cost of increasing the volume of normal tissues exposed to lower doses. Some authors have estimated that IMRT may increase the risk of a second cancer by a factor of 1.2-8 due to both the elevated integral dose to normal tissue and its dose distribution [33,34]. However, other authors have found that the integral dose to non-targeted tissues is relatively unchanged by IMRT and may even be reduced. So, Parker at al. reported a lower integral dose with IMRT than with conventional technique for craniospinal irradiation [11]. Others have observed lower scattered dose with HT compared with other photon IMRT techniques [35]. On the other hand, some authors have found that the integral dose cannot be considered as a good predictor for radiocarcinogenesis [36]. Since the process of radiocarcinogenesis is not yet fully understood, and a quantitative risk assessment still has a lot of uncertainties [37], in absence of an accurate risk model, prospective recording of dosimetrical data seems necessary to evaluate the impact of these novel methods.

The analysis of published series proves that IMRT and HT can be a good alternative for the administration of radiation therapy in pediatric population. These techniques allow good protection of OARs as well as local control rates. These preliminary results should be confirmed in further clinical studies aimed to evaluate the long-term results of HT treatment.

## Conclusion

HT is clinically and technically efficient and feasible technique for the treatment of childhood malignancies. It is associated with an acceptable rate of acute toxicity. A longer follow-up is needed to evaluate the long-term clinical effectiveness and dosimetric advantages of HT over conventional radiotherapy techniques in the treatment of pediatric malignancies.

## Competing interests

Latifa Mesbah, Immacolata Marrone and Sergey Usychkin had financial support from the Grupo IMO Foundation

## Authors' contributions

LM, RM, IM, FM, CM patients data collection, processing and draft of manuscript. SU patient data collection, processing, statistical analysis and elaboration of manuscript final version, LM statistical analysis, RF, GG, CB study design, coordination of data processing. HM study design, coordination, elaboration of manuscript final version.

All authors read and approved the final manuscript.
